# Magnetotransport and conductivity mechanisms in Cu_2_ZnSn_x_Ge_1−x_S_4_ single crystals

**DOI:** 10.1038/s41598-018-35497-y

**Published:** 2018-11-30

**Authors:** Erkki Lähderanta, Elena Hajdeu-Chicarosh, Maxim Guc, Mikhail A. Shakhov, Ivan Zakharchuk, Ivan V. Bodnar, Ernest Arushanov, Konstantin G. Lisunov

**Affiliations:** 1 Department of Physics, Lappeenranta University of Technology, PO Box 20, FIN-53851 Lappeenranta, Finland; 2 Institute of Applied Physics, Academiei Str. 5, MD-2028 Chisinau, Republic of Moldova; 3 Ioffe Institute, Politehnicheskaya Str. 26, St. Petersburg, 194021 Russian Federation; 4 Department of Chemistry, Belarusian State University of Informatics and Radioelectronics, P. Brovki Str. 6, Minsk, 220013 Belarus

## Abstract

Resistivity, ρ(*T*), and magnetoresistance (MR) are investigated in the Cu_2_ZnSn_x_Ge_1−x_S_4_ single crystals, obtained by the chemical vapor transport method, between *x* = 0–0.70, in the temperature range of *T* ~ 50–300 K in pulsed magnetic field of *B* up to 20 T. The Mott variable-range hopping (VRH) conductivity is observed within broad temperature intervals, lying inside that of *T* ~ 80–180 K for different *x*. The nearest-neighbor hopping conductivity and the charge transfer, connected to activation of holes into the delocalized states of the acceptor band, are identified above and below the Mott VRH conduction domain, respectively. The microscopic electronic parameters, including width of the acceptor band, the localization radius and the density of the localized states at the Fermi level, as well as the acceptor concentration and the critical concentration of the metal-insulator transition, are obtained with the analysis of the ρ(*T*) and MR data. All the parameters above exhibit extremums near *x* = 0.13, which are attributable mainly to the transition from the stannite crystal structure at *x* = 0 to the kesterite-like structure near *x* = 0.13. The detailed analysis of the activation energy in the low-temperature interval permitted estimations of contributions from different crystal phases of the border compounds into the alloy structure at different compositions.

## Introduction

In the last years, utilization of the Cu-based group I_2_-II-IV-VI_4_ chalcogenide semiconductors became one of the leading streams in the development of the low-cost thin film solar cells. Different stages have been overcame, and the recent one is based on a partial substitution of different cations in the standard Cu_2_ZnSnS_4_ (CZTS) compound^1–4^. This approach was found to lead to a substantial decrease of the detrimental defects in CZTS, increasing the device efficiency^1–4^. One of the most discussed elements to be add in CZTS is germanium, replacing partially tin. In this framework, several papers demonstrating a positive Ge effect on the solar cell efficiency have appeared recently^4–8^. In addition, the Cu_2_ZnSn_x_Ge_1−x_S_4_ (CZTGS) solid solutions exhibit an increase of the band gap, *E*_g_, with increasing Ge concentration, reaching the values of *E*_g_ up to ~2.3 eV for the pure Cu_2_ZnGeS_4_ (CZGS) compound^9,10^. This allows fine band gap tuning in the CZTGS solid solutions^6,11–13^. Such effect was found to be interesting for the multi-junction solar cells, where CZTGS could be used as a top solar cell^14^.

In addition to photovoltaics, the structural and optical properties of CZTGS^11,13,15–17^, as well as the vibrational properties of this material^16–19^ have been studied. On the other hand, investigations of the electronic transport in CZTGS solid solutions are lacking up to date. The only available data have been obtained in the pure CZTS^20–23^ and CZGS^24,25^ border compounds. The latter was found to be crystalized in the kesterite (KS)^26^ and wurtzstannite (WS)^27^ types of structure, and the electronic properties were studied for each type of CZGS^24,25^. Both CZTS and CZGS compounds exhibit similar activated character of the temperature dependence of resistivity, including the Mott variable range hopping (VRH) conduction within a wide temperature range^22–25^. However, the behavior of magnetoresistance (MR) in these compounds is different, being completely positive (pMR) in CZTS^22^ and possessing a negative contribution (nMR) in CZGS^24^. Therefore, similar activated conductivity with the Mott VRH conduction within a broad temperature range is expectable for the CZTGS solid solutions, too.

In the present work, investigations of the resistivity, ρ(*T*), and MR have been performed in Cu_2_ZnSn_x_Ge_1−x_S_4_ with different *x* or Sn/(Sn + Ge) ratio values. The purpose is to establish the conductivity mechanisms in various temperature intervals. Second goal of our work is to determine important microscopic electronic parameters of the material, depending on *x*. Such dependences are expected to be sensitive to crystal structure details and may yield valuable information on the phase content of the investigated material.

## Materials and Methods

Single crystals of the Cu_2_ZnSn_x_Ge_1−x_S_4_ solid solutions with *x* = 0–0.70 were grown by a chemical vapor transport method, with preliminary synthesis of the constituent elements in the vertical two zone furnace. The synthesized ingot was grinded and placed in the evacuated ampoule together with 5 mg/cm^3^ of iodine, which was used as a transporter. The evaporation temperature was about 80 K below the growing temperature (~ 970 K). More details about the growing process can be found elsewhere^11,13,18^. As a result, four samples with different Ge/Sn ratio (*x* = 0, 0.13, 0.51 and 0.70) were selected for the subsequent analysis.

The structural and Raman scattering analyses of the samples taken from the same set have been performed previously, and the results can be found in ref.^18^. The structural analysis showed that all samples were crystalized in the tetragonal lattice, permitting exclusion of the WS phase. However, the results of ref.^18^ did not yield a certain structural type of our samples, namely the KS or stannite (SN) one, which is a well-known problem of the Cu-Zn based quaternaries^28–30^. On one hand, the Raman scattering data could help to solve this problem, since an additional A-symmetry peak appears in the KS type materials compared to SN^26^. On the other hand, such additional Raman peak was significantly smaller comparing to other peaks in all analyzed samples^18,26^. From these results, it is not possible to exclude the existence of either KS or SN type structure in our samples. Additionally, as expected for single crystals, a good crystalline quality and absence of any secondary phases were found^18^.

Chemical composition of selected samples was obtained using the energy dispersive X-ray microanalysis (EDAX). The composition of all samples was measured at least in five points, and the mean values are collected in Table 1. Here, we found that the real Sn/(Sn + Ge) ratio is quite close to the initial. In turn, the ratio of all cations is also quite close to the stoichiometric values (see three last columns in Table 1).

**Table 1:** Chemical composition of the investigated Cu_2_ZnSn_x_Ge_1−x_S_4_ samples.

*x*	Cu (at. %)	Zn (at. %)	Sn (at. %)	Ge (at. %)	S (at. %)	Cu/(Zn + Sn + Ge)	Zn/(Sn + Ge)	Cu/Zn
0.00	25.04	12.10	0.00	11.87	50.99	1.04	1.02	2.07
0.13	25.38	12.01	1.50	10.03	51.08	1.08	1.04	2.11
0.51	27.05	12.96	6.44	6.21	47.34	1.06	1.02	2.09
0.70	23.94	12.39	8.64	3.75	51.28	0.97	1.00	1.93

The hot probe method, addressed to thermopower measurements, demonstrated the *p*-type conductivity in all the investigated CZTGS samples. The resistivity and MR were measured with a standard dc method using six indium contacts. A pulsed magnetic field (PMF) was applied for the MR measurements, and the sample temperature was controlled using a nitrogen-filled cryostat. A dual compensation method, including the hardware and the software component, was used in order to avoid any induced voltage pertinent of the PMF measurements. More details about the PMF measurement procedure and the installation parameters could be found elsewhere^22^.

## Results and Discussion

### Experimental results

**Figure 1:**
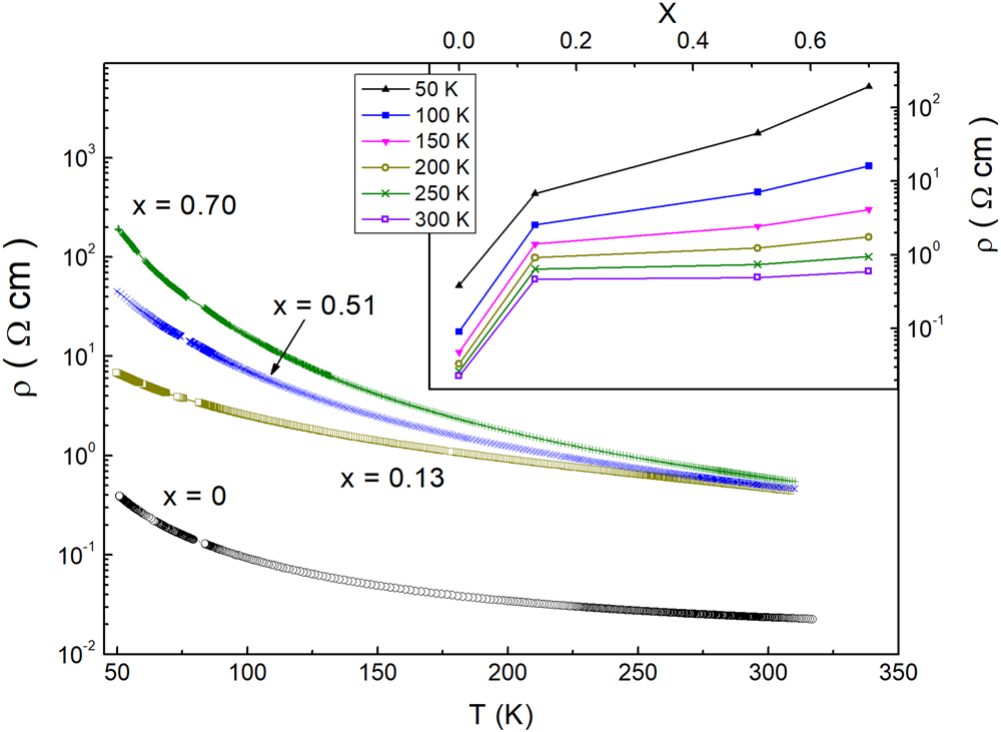
Temperature dependence of the resistivity in the investigated CZTGS samples. Inset: The plots of ρ vs. *x* at different temperatures. The lines simply connect the experimental data points.

As can be seen in Fig. 1, the dependence of ρ(*T*) is activated in all samples, weakening between *x* = 0 and 0.13 and strengthening with increasing *x* between 0.13–0.70. Such behavior is attributable to a different proximity of samples to the metal-insulator transition (MIT) at different *x*, which will be verified later. In addition, the dependence of ρ on *x* is also different within two intervals of the composition. This is visible in the inset to Fig. 1, where ρ(*x*) exhibits a sharp increase between *x* = 0–0.13 and weakening with further increasing *x* at any *T*, especially at high temperatures. Preliminarily, such behavior is attributable to a possible existence of the SN phase at *x* = 0, which is expectable in the pure CZGS compound, as well as the KS phase (see Section “Materials and Methods”). However, the SN phase is usually less stable than the KS phase^29,31^. So, in CZTGS it becomes unstable even at a small Sn content, transforming probably into a KS-like phase already at *x* = 0.13. Therefore, further evolution of the material properties should be smoother, reflecting only differences between the KS phases of the border compounds. This agrees with weakening of the ρ(*x*) dependence above *x* = 0.13. Such conjecture, addressed to a deeper discussion of the resistivity depending on *T* and *x*, will be verified below, too.

**Figure 2:**
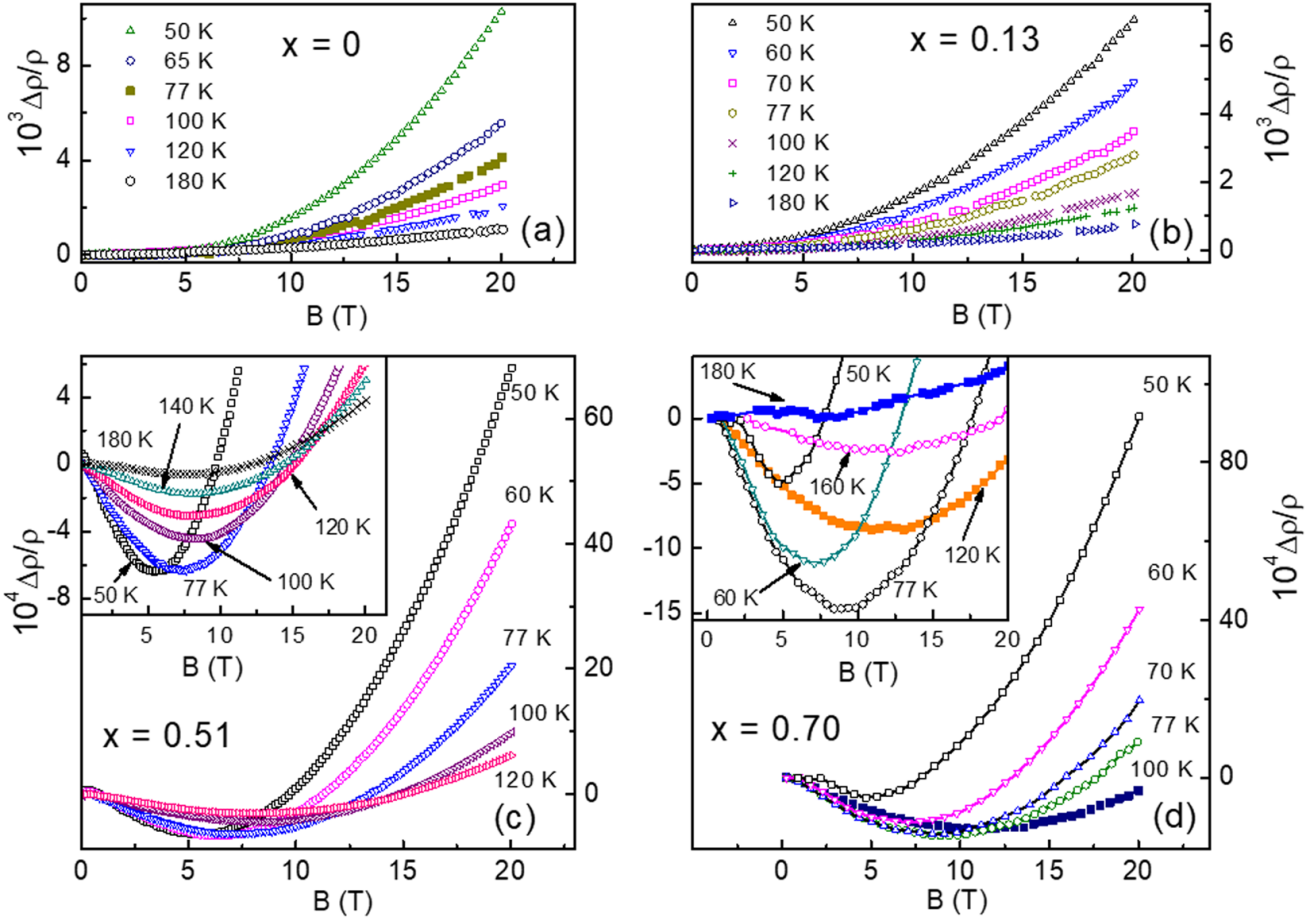
The dependences of Δρ/ρ on *B* for *x* = 0 (**a**), 0.13 (**b**), 0.51 (**c**) and *x* = 0.70 (**d**).

MR, Δρ/ρ ≡ [ρ(*B*) − ρ(0)]/ρ(0), is positive (pMR) almost everywhere in samples with *x* = 0 and 0.13, as visible in Fig. 2(a,b), respectively. A small overall negative (nMR) effect with the maximum absolute values of ~4 × 10^−5^ at *T* = 65 K and ~3 × 10^−6^ at *T* = 77 K is observed at *x* = 0 only in fields below *B* ~3 − 4 T, decreasing rapidly with *B* and *T*. On the other hand, in samples with *x* = 0.51 and especially *x* = 0.70 the behavior of MR is quite different, as can be seen in Fig. 2(c,d), respectively. Namely, in the former pMR is observed in high fields at any temperature (Fig. 2(c)), whereas the nMR contribution appears with lowering *B* already at 180 K, which is visible in the inset to Fig. 2(c). The overall nMR effect is much stronger at *x* = 0.51 than at *x* = 0, attaining a maximum up to 6.6 × 10^−4^. Moreover, below *B* ~ 5 T the dependence of nMR on *T* between 50–77 K is quite weak, shifting only the position of the nMR minimum to higher fields. In addition, the shape of the MR curves is different within various temperature intervals, exhibiting a substantial broadening above 77 K. The behavior of MR in the sample with *x* = 0.70 is even more complicated, exhibiting an increase of the nMR contribution with *T* increasing between 50–77 K, as evident in the inset to Fig. 2(d). Additionally, the overall nMR effect is higher at *x* = 0.70 than at *x* = 0.51, reaching the value of 1.5 × 10^−3^. Generally, such features of nMR are quite uncommon for conventional doped semiconductors. However, they have been already observed in the pure CZGS^24^ and, partially, in the Cu_2_ZnSn_x_Ge_1−x_Se_4_ alloys^32^.

### Temperature dependence of the resistivity at B = 0

In kesterites and related compounds, different mechanisms of the charge transfer have been observed within different temperature intervals Δ*T*^22–25,32–41^. These include (i) the nearest-neighbor hopping (NNH) at *T* lying within Δ*T*_n_, (ii) the Mott VRH hopping inside the interval Δ*T*_M_ and (iii) thermal activation of charge carriers into an interval of delocalized states in the acceptor band at temperatures within Δ*T*_a_^42–44^. In all cases above, ρ (*T*) is given by a universal expression,

1 $${\rm{\rho }}(T)={{\rm{\rho }}}_{0}(T)\exp \,[{(\frac{{T}_{0}}{T})}^{1/p}],$$

where *p* = 1 for the cases (i) and (iii), and *p* = 4 for the mechanism (ii). The prefactor ρ_0_(*T*) ∝ *T*^−1^ for the NNH conduction, ρ_0_(*T*) ∝ *T*^−1/4^ for the Mott VRH conduction, and ρ_0_ = const for the last out of the cases above. For the case (ii), the exponential factor of Eq. (1) is governed by the characteristic temperature, *T*_0_ = β/[*k*_B_ *g*(μ) *a*^3^], where β = 21, *k*_B_ is the Boltzmann constant, *g*(μ) is the density of states (DOS) at the Fermi level, μ, and *a* is the localization radius of charge carriers^42–44^. For the cases (i) and (iii), instead of *T*_0_ the corresponding activation energies, *E*_n_ = *k*_B_*T*_0_ and *E*_a_ = *k*_B_*T*_0_ are used, respectively. Here, *E*_a_ = |*E*_c_ − μ|, where *E*_c_ is the mobility edge of the acceptor band (AB) and μ is the Fermi energy^42,43^.

**Figure 3:**
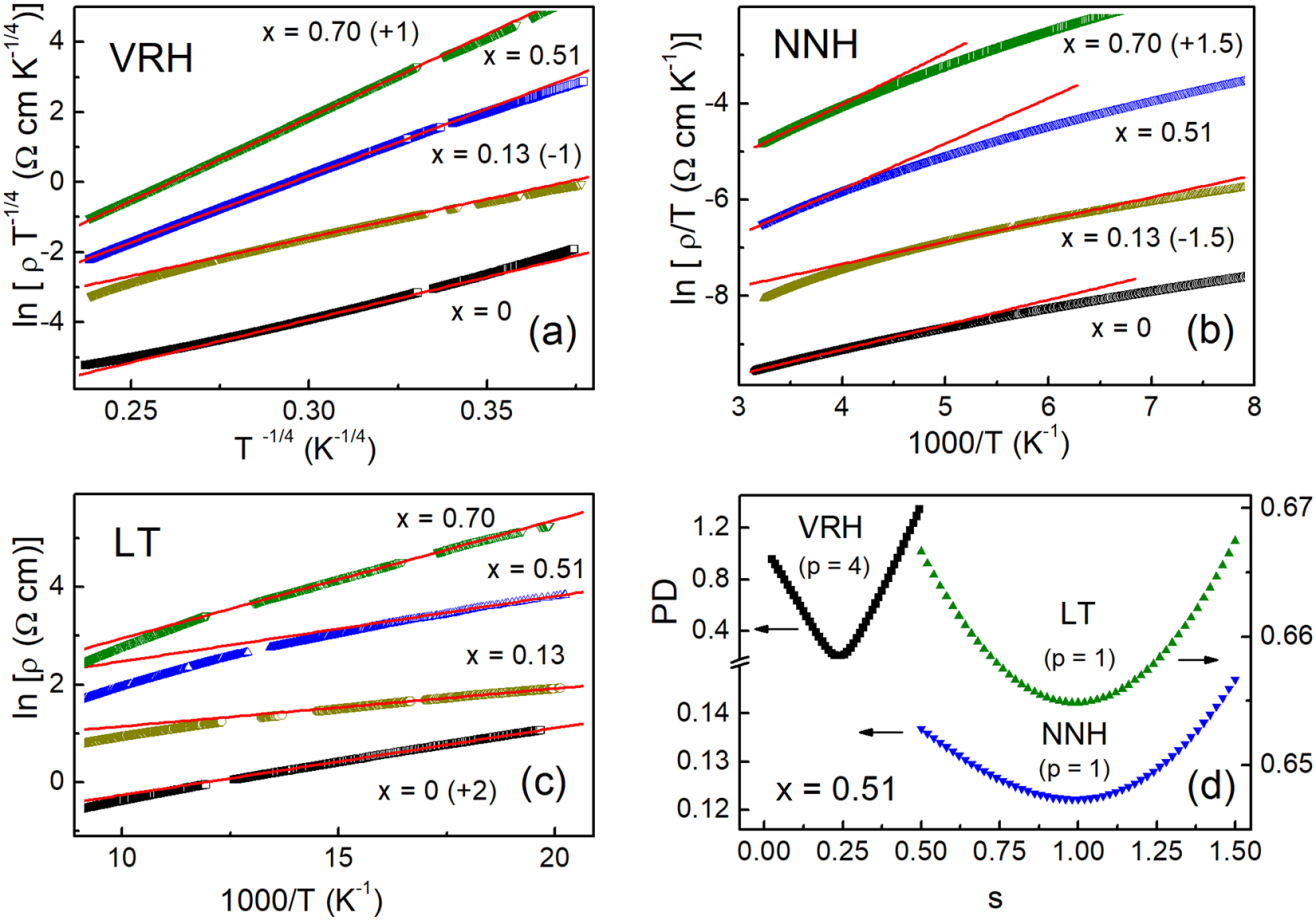
The plots of ln(ρ*T*^−1/4^) vs. *T*^−1/4^ (**a**), the plots of ln(ρ/*T*) vs. 1000/*T* (**b**), the plots of ln(ρ) vs. 1000/*T* (**c**), and the plots of PD vs. *s* (**d**). Some of the plots are shifted along the vertical axes by the values, given in parenthesis, for convenience. The lines are linear fits.

**Figure 4:**
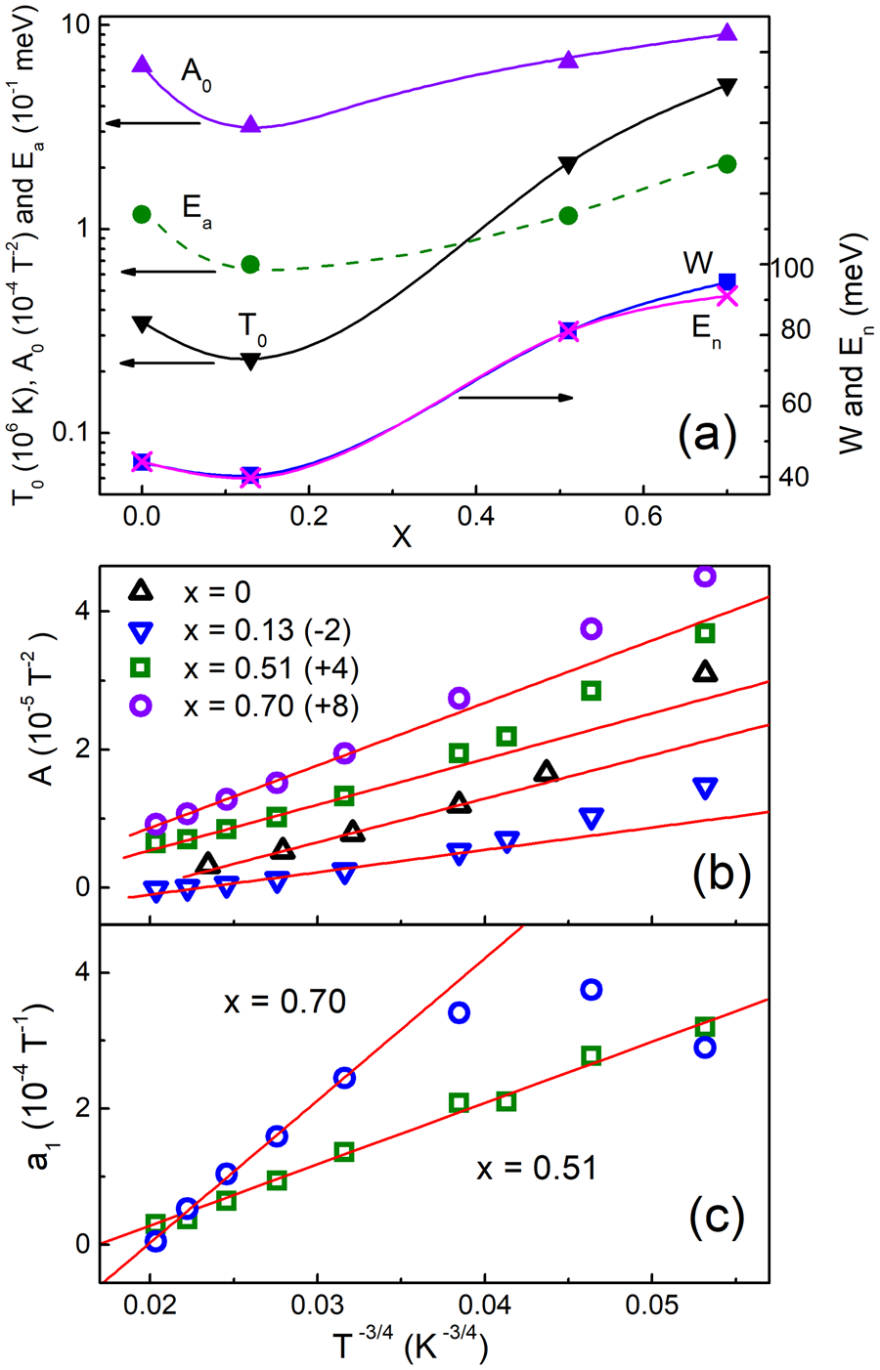
The plots of *T*_0_, *A*_0_, *E*_a_, *W* and *E*_n_ vs. *x*. The lines are to guide the eye (**a**). The dependences of *A* on *T*^−3/4^ for different *x*. Some of the data are shifted along the vertical axis by the values, given in parenthesis, for convenience. The lines are linear fits (**b**). The plots of *a*_1_ vs. *T*^−3/4^. The lines are linear fits (**c**).

In quaternary chalcogenides, the widest temperature interval belongs to the Mott VRH conduction^22–25^. Therefore, here we start the analysis of ρ(*T*) by searching the interval Δ*T*_M_ and determination of *T*_0_. As follows from Fig. 3(a), ρ(T) can be linearized according to Eq. (1) at *p* = 4 within intervals of Δ*T*_M_ (see Table 2). The values of *T*_0_ have been obtained from the slopes of the plots in Fig. 3(a) and are shown in Fig. 4(a) as a function of *x*, along with the corresponding width of the AB, *W*, found with the expression *W* ≈ 0.5 *k*_B_ (*T*_v_^3^ *T*_0_)^1/4^ ^34,35^. Here, *T*_v_ is the onset of the VRH conduction on cooling (i. e. the right edge of the Δ*T*_M_ intervals in Table 2).

**Table 2:** The temperature intervals Δ*T*_n_, Δ*T*_M_ and Δ*T*_a_ of the NNH, VRH and LT conductivities, respectively, and the values of the effective mass, m* (in units of *m*_0_).

*x*	Δ*T*_n_ (K)	Δ*T*_M_ (K)	Δ*T*_a_ (K)	m*
0.00	265–310	95–145	50–65	0.49
0.13	170–195	80–150	50–60	0.60
0.51	270–310	95–180	50–60	0.54
0.70	265–310	80–165	55–75	0.51

If μ lies close to the AB edge, the deviations of ρ(*T*) from the Mott law above *T*_v_ can be explained by the transition to the NNH conduction (see above), where *E*_n_ ≈ *W*^44^. As can be seen in Fig. 3(b), for all samples ρ(*T*) can be linearized according to Eq. (1) at *p* = 1 within the intervals Δ*T*_n_ collected in Table 2. The values of *E*_n_, obtained from the linear fit of the plots in Fig. 3(b) within the intervals Δ*T*_n_, are displayed in Fig. 4(a) along with the *W* data, demonstrating a complete agreement with the latter at an error given by the size of the data points.

In turn, deviations of ρ(*T*) in the low-temperature (LT) intervals, Δ*T*_a_, lying below Δ*T*_M_, are attributable to the case (iii) above. Indeed, the plots in Fig. 3(c) demonstrate a good linearity within intervals Δ*T*_a_ evidently below the temperatures of the Mott VRH conduction region (see Table 2) and yielding the data of *E*_a_, displayed in Fig. 4(a).

Finally, the values of *s* = 1/*p* were obtained with a “percentage deviation” (*PD*) method, where *PD* represents the relative difference between the experimental and calculated resistivity data^45^. This was done by minimizing *PD* inside the intervals Δ*T*_M_, Δ*T*_n_ and Δ*T*_a_, at different values of *s* (see refs^22,34^ for details). We found a complete agreement between the values of *s* and 1/*p* for all samples. An example of such procedure is displayed in Fig. 3(d) for *x* = 0.51.

### Analysis of the magnetoresistance

In the domain of the Mott VRH conduction, pMR is connected mainly to shrinkage of the impurity wave functions by the magnetic field^44^. In particular, this mechanism is the only one leading to pMR in weak magnetic fields of λ ≫ *a*, where λ = [ħ/(*eB*)]^1/2^ is the magnetic length, ħ is the Planck constant and *e* is the elementary charge. Here, pMR is given by the expression

2 $$\mathrm{ln}[\frac{{\rm{\rho }}\,(T,B)}{{\rm{\rho }}\,(T,0)}]=A(T){B}^{2},$$

where *A*(*T*) = *A*_0_ *T*^−3/4^, A_0_ = *t*(*e*^2^ *a*^4^/ħ^2^)*T*_0_^3/4^ and *t* = 5/2016^44^. Because the overall nMR effect at *x* = 0 is small (see Section “Experimental results”), Eq. (2) can be utilized for the analysis of MR in this sample at least for the strong enough *B* values, where the nMR contribution is expected to be negligible. In the sample with *x* = 0.13 the overall nMR effect is not observed, so that Eq. (2) can be utilized without any restrictions, excluding only that of λ ≫ *a* mentioned above. However, at *x* = 0.51 and 0.70 the nMR contribution is increased to be taken into account more carefully.

As has been demonstrated recently^24,32^, the most probable mechanism of nMR in CZGS is connected with quantum interference effects in the Mott VRH conduction interval^46–50^. Therefore, we can utilize this mechanism for interpretation of nMR in our CZTGS alloys, too, especially taking into account the close similarity of the MR behavior in both materials, mentioned in Section “Experimental results”. Accordingly, for not too low magnetic fields nMR contribution can be written as (Δρ/ρ)_n_ = −*a*_1_(*T*)*B*, where *a*_1_(*T*) ∝ *T*^*−*3/4^ similar to *A*(*T*) above^49^. Taking into account the smallness of MR in our samples, providing a good accuracy for the approximative relation of ln[ρ(*T*,*B*)/ρ(*T*,0)] ≈ Δρ/ρ, we can express pMR with Eq. (2) as (Δρ/ρ)_p_ = *A*(*T*)*B*^2^. Therefore, using the conventional expression of Δρ/ρ = (Δρ/ρ)_n_ + (Δρ/ρ)_p_, one finds the equation

3 $${\rm{\Delta }}{\rm{\rho }}/{\rm{\rho }}+{a}_{{\rm{1}}}(T)B=A(T){B}^{2},$$

available for the analysis of MR in samples with *x* = 0.51 and 0.70, which can be performed here with the method, applied recently for CZGS^24^. Namely, the values of *a*_1_(*T*) can be found by plotting the left-hand side of Eq. (3) vs. *B*^2^ and varying *a*_1_ up to reach the best linearization of the plots. The latter can be done by minimizing the standard deviation (SD) of the plots, under an additional condition for the linear parts of the plots to pass through the origin^24^.

**Figure 5:**
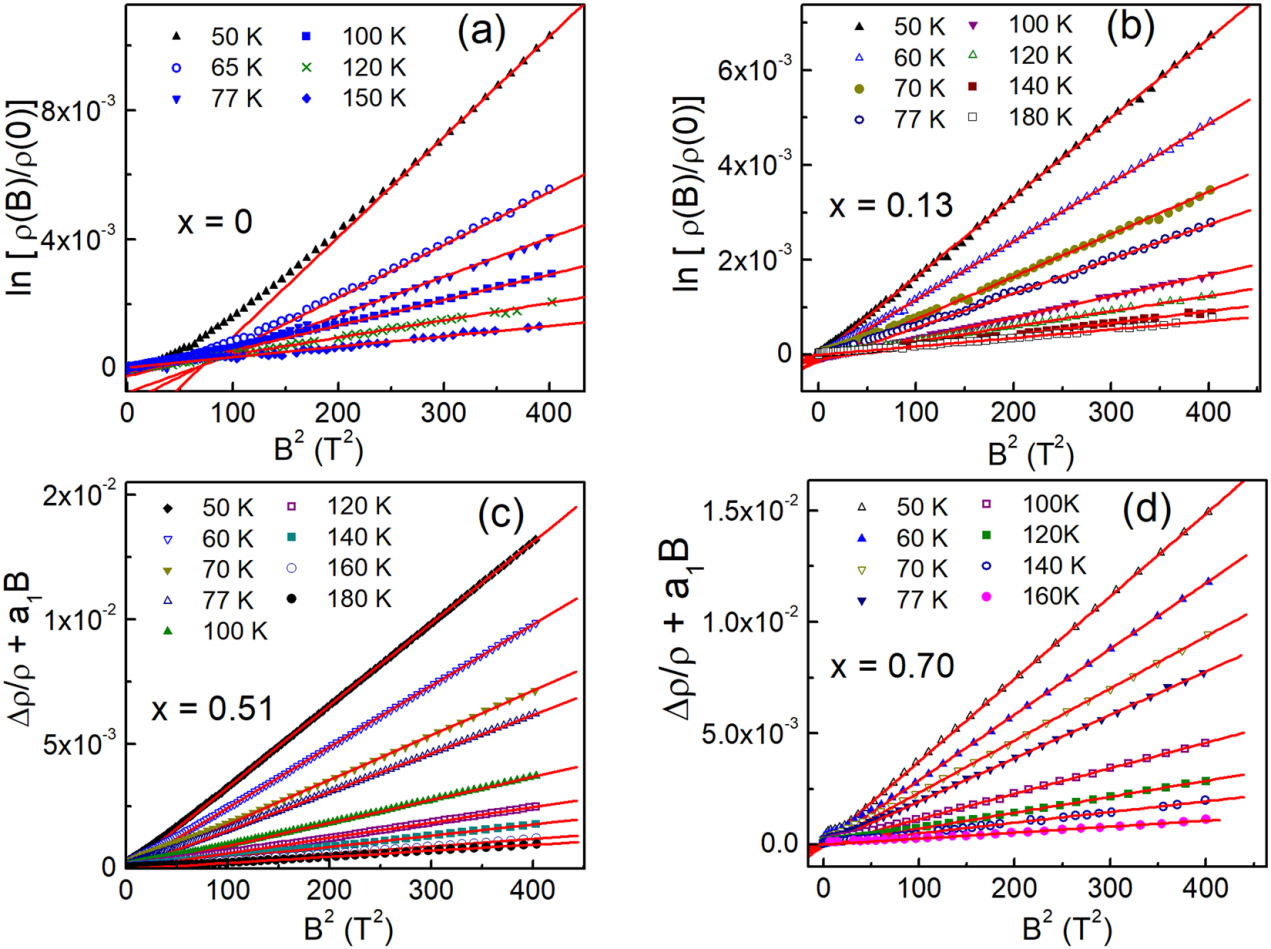
The plots of ln[ρ(*B*)/ρ(0)] vs. *B*^2^ for *x* = 0 (**a**) and *x* = 0.13 (**b**). The plots of Δρ/ρ + *a*_1_*B* vs. *B*^2^ for *x* = 0.51 (**c**) and *x* = 0.70 (**d**). The lines are linear fits.

Then, the values of *A*(*T*) can be found from the slope of the plots shown in Fig. 5. Namely, this is provided by good linearity of these plots, excluding only the cases of *T* = 50 and 65 K in the sample with *x* = 0, deviating from the linearity with decreasing *B*, as seen in Fig. 5(a), due to a small nMR contribution (see Section “Experimental results”). However, this does not look to be a big problem, because the linear parts are still broad enough in the scale of Fig. 5(a) and, additionally, these temperature points lie outside the Mott VRH interval (cf. Table 2).

Eventually, the data of *A*(*T*) are plotted vs. *T*^−3/4^ in Fig. 4(b), exhibiting a good linearity within the whole interval Δ*T*_M_ of the Mott VRH conduction (cf. Table 2). A small deviation of the data point for *x* = 0.5 from the perfect linearity takes place only at *T* = 180 K (or *T*^−3/4^ ≈ 0.02 *K*^−3/4^ in the scale of Fig. 4(b)) lying just on the border of Δ*T*_M_. On the other hand, the stronger deviations of the plots in Fig. 4(b) from the linear behavior takes place in all samples with decreasing *T*, but this occurs already below Δ*T*_M_ (see Table 2). In addition, the dependences of *a*_1_(*T*) ∝ *T*^−3/4^ also take place in both samples with *x* = 0.51 and 0.70, as can be seen in Fig. 4(c), violating only outside the Mott VRH conduction interval at *x* = 0.70.

Hence, in our material both the nMR and the pMR contributions to MR demonstrate the field and temperature dependences, which are in a complete agreement with mechanisms described above. Finally, the data of *A*_0_ have been determined from the slope of the linear parts of the plots in Fig. 4(b) and are displayed in Fig. 4(a).

### Determination of microscopic parameters and analysis of E_a_

**Figure 6:**
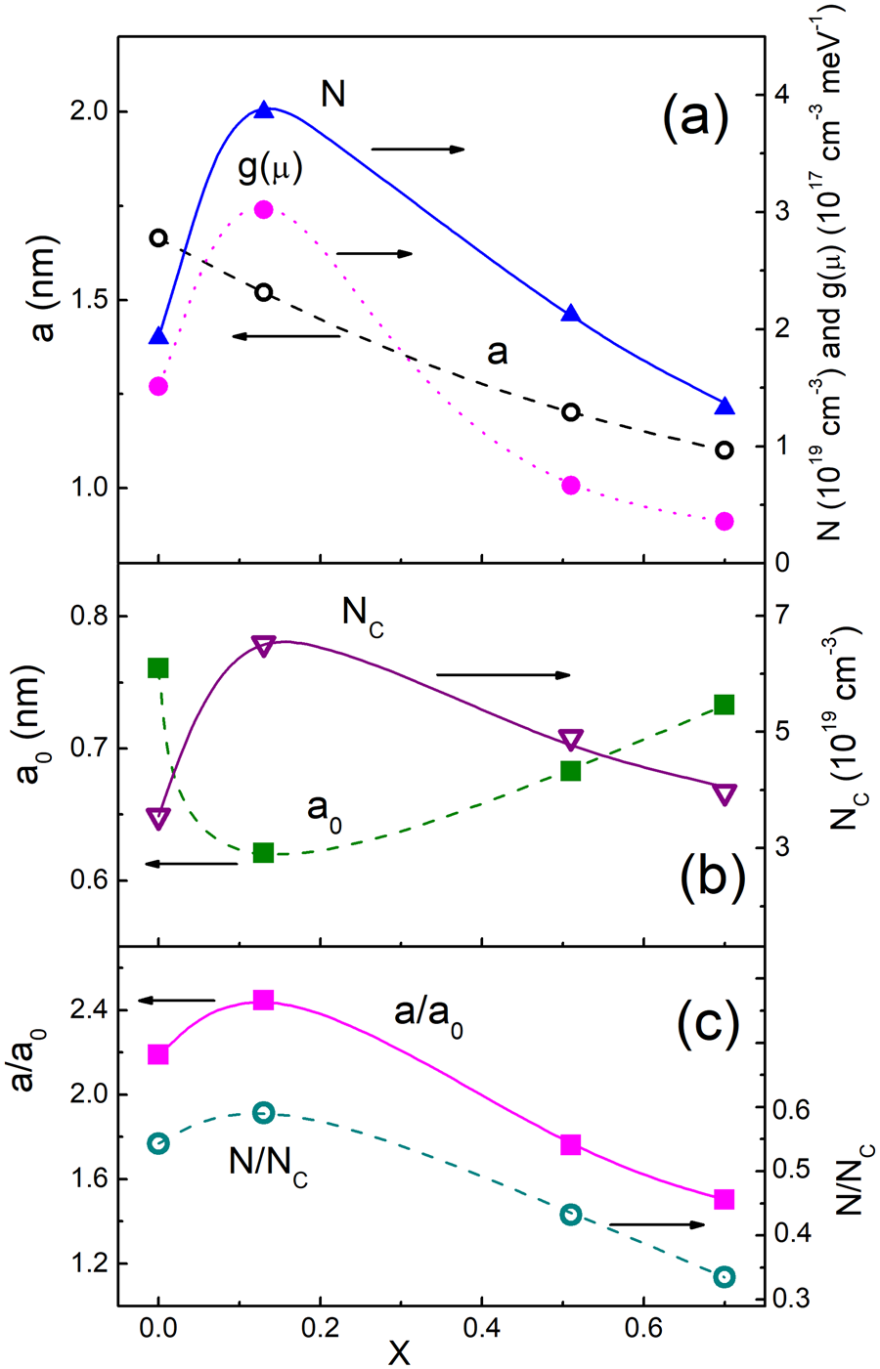
The dependences of *a*, *g*(μ) and *N* on *x* (**a**). The plots of *a*_0_ and *N*_c_ vs. *x* (**b**). The dependences of *a*/*a*_0_ and *N*/*N*_c_ on *x* (**c**). The lines are to guide the eye.

First of all, a pair of the parameters, *a* and *g*(μ), can be found directly with the pair of the corresponding expressions of *T*_0_ and *A*_0_, given in the text below Eqs (1) and (2), respectively. The obtained values of *a* and *g*(μ) are plotted vs. *x* in Fig. 6(a). Next, for the analysis of the *E*_a_ data and determination of further electronic parameters we use a general expression of *a*,

4 $$a={a}_{0}{(1-N/{N}_{C})}^{-v},$$

where *N* and *N*_C_ are the acceptor concentration and the critical concentration of the MIT, respectively^51^. Here, the value of the localization radius far from the MIT, *a*_0_, is usually close to the Bohr radius, *a*_B_ = ħ^2^κ_0_/(*m* * *e*^2^)^44^, where κ_0_ is the dielectric permittivity of the material far from the MIT, m* is the carrier effective mass and ν ≈ 1 is the critical exponent^51^. Another expression of the localization radius, is given by the equation^42,43^.

5 $$a={a}_{{\rm{0}}}{(1-\frac{W+\mu }{W+{E}_{c}})}^{-\nu }.$$

Here, the energy is measured from the center of AB, *E*_A_, which represents the mean energy of the acceptor levels, so that μ < *E*_c_ < 0^24^. Finally, we approximate the DOS of AB with the Gaussian shape, which yields the expression.

6 $$g({\rm{\mu }})=N/({\pi }^{1/2}W)\exp [-{({\rm{\mu }}/W)}^{2}].$$

The subsequent analysis of *E*_a_ can be performed with Eqs (4–6) by assuming the relation *a*_0_ = *a*_B_ at κ_0_ = 7. Indeed, this value of κ_0_ has been deduced from the capacitance spectra of CZTS^52^, whereas the close data of κ_0_ = 6.68 and 6.8 have been predicted with the first-principle calculations for CZGS with the KS and SN structures, respectively^53^. Therefore, the overall variation of κ_0_ with *x* can be neglected, deviating from the value of κ_0_ = 7 only by a few percent. Eventually, we use the universal Mott criterion, *N*_C_^1/3^*a*_B_ ≈ 0.25^42,43^.

**Figure 7:**
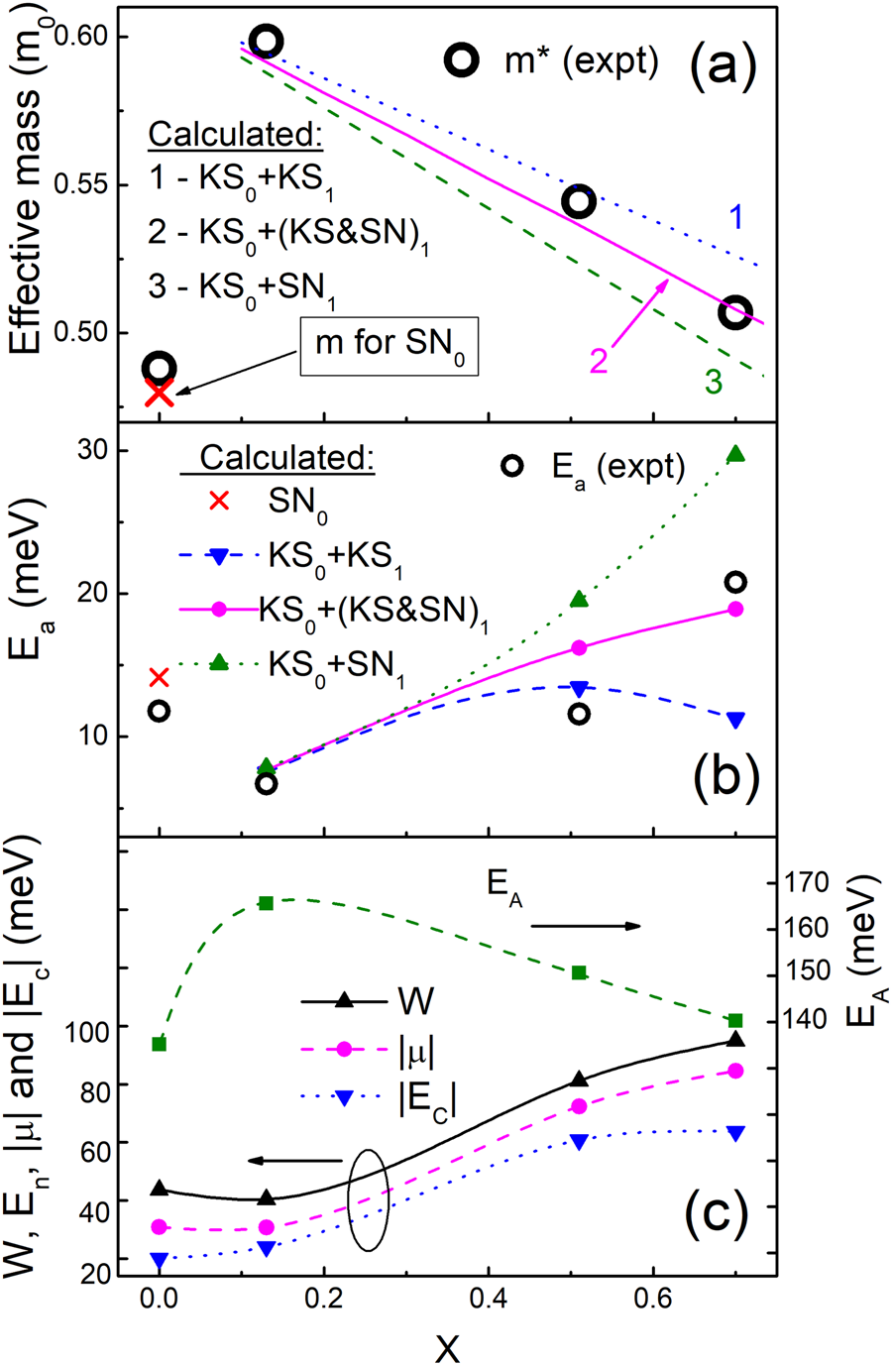
The plots of the experimental effective mass m* vs. *x* (open circles). The lines 1–3 are evaluated as described in the text. The single oblique cross represents the effective mass value for the SN-CZGS phase (**a**). The dependence of the experimental activation energy *E*_a_ on *x* (open circles). The data given by the closed up triangles, closed circles and closed down triangles are evaluated, as described in the text. The lines are spline interpolations of the data points. The single oblique cross represents the *E*_a_ value, calculated with the effective mass of the SN-CZGS structure (**b**). The dependences of *E*_A_, *W*, |μ| and |*E*_c_| on *x*. The lines are to guide the eye (**c**).

Hence, the only unknown parameter, required for calculations of μ, *E*_c_ and, finally, *E*_a_ is the effective mass m*. This parameter can be obtained by an explicit fit of the experimental *E*_a_ data with the expression *E*_a_ = |μ−*E*_c_|, using the following simple procedure: (i) taking a certain m* value, one can evaluate *a*_0_ = *a*_B_ and *N*_C_ with the expression of *a*_B_ above and with the Mott criterion, respectively; (ii) then *N* can be obtained with Eq. (4) by utilization of the *a* data in Fig. 6(a); (iii) the knowledge of the parameters *N* and *g*(μ), where the data of *g*(μ) are displayed Fig. 6(a), too, permits determination of μ with Eq. (6); (iv) then, *E*_c_ can be calculate with Eq. (5); (v) finally, *E*_a_ can be found with the difference of μ and *E*_c_, obtained above. Such procedure has been repeated for each of the *E*_a_ values in Fig. 4(a) by variation of m* up to a complete agreement of the experimental and calculated *E*_a_ values, and the resulting data of m* vs. *x* are presented in Fig. 7(a). For convenience, they are collected also in Table 2.

The obtained m* values can be compared with the calculated data of the effective mass, using the mean values of *m* = (*m*_t_^2^*m*_l_)^1/3^, which determine *a*_B_ in a case of the non-spherical carrier spectrum^44^. Here, *m*_t_ and *m*_l_ is the transversal and the longitudinal effective mass components, respectively, of the holes in the upmost valence band, which have been predicted for CZTS and CZGeS with the first-principle calculations^54^. Hence, using the data of ref.^54^ we find the following values (in units of the free electron mass, *m*_0_): *m*_KS_ (CZGS) = 0.61, *m*_SN_ (CZGS) = 0.48, *m*_KS_ (CZTS) = 0.49 and *m*_SN_ (CZTS) = 0.44, for the KS-CZGS, SN-CZGS, KS-CZTS and SN-CZTS phases, respectively.

The comparison of the data above with the values of m* in Table 2 indicates a close proximity of m* at *x* = 0 to *m*_SN_(CZGS), whereas m* at *x* = 0.13 is much closer to *m*_KS_(CZGS). In turn, for *x* between 0.13 and 0.70 the data of m* are lying between those of *m*_KS_(CZGS) and *m*_KS_(CZTS) or *m*_SN_(CZTS). Then, to account for a gradual variation of the phase content in CZTGS with *x*, we apply the linear dependences of the mean effective mass between *x* = 0.13 and 0.70 in the following forms: *m*(KS_0_ + KS_1_) = *m*_KS_(CZGS) + [*m*_KS_(CZTS) − *m*_KS_(CZGS)]*x*, *m*[KS_0_ + (KS&SN)_1_] =*m*_KS_(CZGS) + {[*m*_KS_(CZTS) + *m*_SN_(CZTS)]/2 − *m*_KS_(CZGS)}*x*, and *m*(KS_0_ + SN_1_) = *m*_KS_(CZGS) + [*m*_SN_(CZTS) − *m*_KS_(CZGS)]*x*. Such dependences covering different possible phases of the border compounds at *x* = 0 and 1 (given above by the subscripts 0 and 1, respectively), including a mixed KS and SN phase denoted here as KS&SN, are plotted in Fig. 7(a) with the straight lines along with the experimental m* data (open circles). The data point for the pure SN-CZGS mass (single oblique cross) is added to Fig. 7(a) for completeness.

As follows from Fig. 7(a), the contribution of the SN-CZTS phase is out of game, because *m*(KS_0_ + SN_1_) (line 3) is close to m* only at *x* = 0.13, where the impacts of any CZTS phases are simply too small. This means that the SN-CZTS phase, being less stable in the pure CZTS compound than the KS-CZTS phase^28–30^, cannot be stabilized even by introduction of Ge. On the other hand, a coincidence of *m*[KS_0_ + (KS&SN)_1_] (line 2) with m* at *x* = 0.70 suggests a more importance of the mixed KS and SN structure of CZTS, contributing to the CZTGS alloy phase content, than that of the pure KS-CZTS phase (line 1 lies clearly above m* at *x* = 0.70). Eventually, both lines 1 and 2 lie equivalently around the data point of m* at *x* = 0.51, which does not permit to make a comprehensive conclusion at this point of the CZTGS alloy, because the mixed (KS&SN)-CZTS phase, generally, cannot coexist with the pure KS-CZTS phase.

On the other hand, the issue above can be clarified by the direct calculation of *E*_a_ vs. *x* with the linear dependences of the effective mass between *x* = 0.13 and 0.70 given above, being performed without any fitting procedure, as displayed in Fig. 7(b). Here, the calculations can be realized only at selected values of *x*, and the lines in Fig. 7(b) are only the spline interpolations of the data evaluated at *x* = 0.13, 0.51 and 0.70, respectively. The calculated value of *E*_a_ at *x* = 0 with *m*_SN_(CZGS) is also presented in Fig. 7(b) with the oblique cross point, exhibiting a reasonable agreement with the experimental *E*_a_ value. The contribution of the SN-CZTS phase (closed up triangles) to *E*_a_ does not exist again, since at *x* = 0.51 and 0.70 the corresponding calculated values lie substantially above both these experimental data points. The calculated *E*_a_ value for the mixed (KS&SN)-CZTS phase (closed circles) lie close to the experimental data point at *x* = 0.70, which confirms the main role of this phase in formation of the alloy structure at this composition, following from Fig. 7(a). On the other hand, the calculated *E*_a_ value for the pure KS-CZTS phase (closed down triangles) satisfies the experimental *E*_a_ value at *x* = 0.51 much better, than for the mixed (KS&SN)-CZTS phase. This reflects the dominating contribution of the pure KS-CZTS phase at *x* = 0.51, which removes the ambiguity at this point in Fig. 7(a).

Finally, the parameters following from the explicit fit of *E*_a_ above, including *N*, *N*_c_ and *a*_0_, as well as the ratios of *N*/*N*_c_ and *a*/*a*_0_, are displayed in Fig. 6(a–c), respectively. In addition, the data of *E*_c_ and μ are given in Fig. 7(c) along with the *W* values. The data of *E*_A_, evaluated with the expression for hydrogenic acceptor level, *E*_A_ = ħ^2^/(2*m* * *a*_0_^2^)^44^, are presented in Fig. 7(a), too.

## Discussion

First, the conductivity mechanisms have been determined within temperature intervals Δ*T*_n_, Δ*T*_v_ and Δ*T*_a_ with two different methods, including linearization of the ρ(*T*) data in Fig. 3 (a–c) and application of the percentage deviation method in Fig. 3 (d). The results obtained with both methods are mutually consistent. This provides evidence for the NNH and VRH charge transfer within the temperature intervals Δ*T*_n_ and Δ*T*_v_, respectively. A special attention has been paid to the LT conduction mechanism, characterized by the activation energy *E*_a_.

Namely, we have found that the values of *E*_a_ obtained in the LT region of Δ*T*_a_ above, exhibit a reasonable agreement with those calculated with the expression of *E*_a_ = |μ − *E*_c_| in previous Section. This gives a strong support to the nature of the low-temperature activated conductivity mechanism, acting below the Mott VRH conduction temperatures within Δ*T*_M_ (Table 2), and connected with thermal activation of holes into the region of the delocalized states of AB (see previous Section). It is important to note, that *E*_a_ appears to be rather sensitive to the details of the CZTGS alloy structure, which is evident from previous Section and permits estimation of contributions of the different CZGS and CZTS phases to the CZTGS alloy structure. In particular, a steep increase of ρ(*x*) between *x* = 0 and 0.13, ascribed tentatively in Section “Experimental results” to the possible transition from the SN to KS phase of CZGS and followed by a weaker ρ(*x*) dependence between *x* = 0.13–0.70, finds a convincing explanation by the analysis of *E*_a_(*x*).

Next, the joint analysis of the ρ(*T*) and pMR data permitted determination of such important microscopic parameters as *W*, *E*_C_ and *g*(μ), characterizing the energy spectrum of the holes in AB, as well as those of *a*, *N*, *N*_C_ and *E*_A_. As can be seen in Figs 4, 6 and 7, the majority of these parameters (excluding only *E*_C_) exhibit extremums near *x* = 0.13, similar to those of ρ(*x*) and *E*_a_(*x*) in Figs 1 and 7(b), respectively. This suggests a similar reason for such extremums, reflecting the transition above, too. On the other hand, the cation ratios in the analyzed samples are also not constant and reaches the maximum for the sample with *x* = 0.13 (see the values in the last three columns of Table 1). Taking into account the overwhelming effect of Cu_Zn_ defects to the AB formation^55–58^, the influence of Cu/Zn ratio should be always considered in the quaternary compounds containing these cations.

As can be seen in Fig. 6(c), samples with different *x* are characterized by various values of *N*/*N*_C_ and *a*/*a*_0_, implying their different proximity to the MIT according to Eq. (4). This supports completely the corresponding conjecture formulated in the beginning of Section “Experimental results”.

Finally, as follows from Fig. 2 and the corresponding discussion in Section “Experimental results”, nMR at *x* = 0 is quite small, whereas at *x* = 0.13 it is even absent. At the same time, nMR for *x* = 0.51 becomes important, and at *x* = 0.70, closest to CZTS, nMR attains the maximum value. The reason to such behavior is that nMR due to the quantum interference effects in the VRH conduction domain is highly sensitive to the degree of disorder^46^, which is indicated by the *W* value. As can be seen in Fig. 4(a), *W* in samples with *x* = 0 and 0.13 is substantially smaller, than in those with *x* = 0.51 and 0.70, which is in line with the strong sensitivity of nMR to the disorder, mentioned above.

## Conclusions

We have investigated the resistivity and the magnetoresistance in the Cu_2_ZnSn_x_Ge_1−x_S_4_ single crystals. The analysis of the ρ(*T*) and MR data permits identification of the conductivity mechanisms within different temperature intervals between *T* ~ 50–300 K. Namely, at high temperatures the conduction is realized by the NNH charge transfer, followed by the Mott VRH conduction with decreasing temperature, and eventually by the activation of holes into the interval of delocalized states of the acceptor band, observed within the lowest temperature interval. Detailed analysis of the activation energy in the latter interval gives evidence for the transition of CZTGS from the SN phase to a KS phase with increasing *x* between 0–0.13, and the subsequent smooth evolution of the material within a KS-like structure. The contributions of different phases, pertinent to the border compounds, to the mixed-phase alloy state have been estimated. The values of the important microscopic parameters of the material, including width of the acceptor band, the localization radius and the density of states at the Fermi level, as well as the acceptor concentration have been determined. All the parameters above exhibit extremums near *x* = 0.13, which is connected mainly to the crystalline structure phase transformation near this point, as well as to the possible influence of the Cu/Zn ratio.
